# Real-Time Quantitative PCR: Primer Design, Reference Gene Selection, Calculations and Statistics

**DOI:** 10.3390/metabo13070806

**Published:** 2023-06-28

**Authors:** Zhiwei Chen, Nigel G. Halford, Chenghong Liu

**Affiliations:** 1 Shanghai Key Laboratory of Agricultural Genetics and Breeding, Biotechnology Research Institute, Shanghai Academy of Agricultural Sciences, Shanghai 201106, China; liuchenghong@saas.sh.cn; 2 Rothamsted Research, Harpenden AL5 2JQ, Hertfordshire, UK; nigel.halford@rothamsted.ac.uk

**Keywords:** quantitative real-time PCR, relative gene expression, normalized relative quantity, PCR efficiency

## Abstract

Real-time quantitative PCR is a technique that can measure the content of the target nucleic acid sequence of interest in a given sample. It is mainly divided into absolute and relative quantitative methods. The relative quantification is mainly used in gene expressions for functional genomic and transcriptome studies. However, to use this technology accurately, there are some key points to master. First, specific primers need to be designed to ensure amplification of the gene of interest (GOI). Second, the appropriate reference gene or reference gene combination has to be selected. Finally, scientific gene expression level calculations and statistics are required to obtain accurate results. Therefore, this work proposes a workflow for relative quantitative PCR and introduces the relevant points so that beginners can better understand and use this technology.

## 1. Introduction

Real-time quantitative PCR, also called quantitative PCR (qPCR), combines PCR and fluorescent dyes to monitor template amplification with high sensitivity and specificity. At present, qPCR has been widely used in gene expression analyses, genotyping, microbial quantification, virus detection, and more [1,2]. There are two main methods of qPCR for gene quantification and expression analyses, namely absolute and relative quantification. Here, this review mainly focuses on the relative quantification method.

As we know, the formula of 2^−ΔΔCt^ is commonly used to calculate the relative quantification of gene expression. However, when using this formula, there are some limitations for “2” calculated by formula “1 + e (PCR amplification efficiency)”, that is, the PCR amplification efficiencies of all primers for both the reference gene and the gene of interest (GOI) should be 100% or close to 100% at the same time, or at least between 90% and 110% (or 105%) [2,3]. As a result, when designing primers, we need to test their amplification efficiencies before using them in an experiment, which increases the workload and limits the number of primers that can be used. In the worst case, the amplification efficiency is arbitrarily taken as two. In addition, using this formula, the relative gene expression levels in the control samples are all equal to one, which is not scientific in statistics. At the same time, it is also easy to exaggerate the role of low-abundance expression genes. In other words, 2^−ΔΔCt^ represents a ratio between samples and does not show the relative expression of target gene to the reference gene(s), so the importance of target genes cannot be assessed. For example, the ratio of 0.01/0.00001 is much larger, but, in fact, 0.01 is a very low expression level relative to the reference gene(s), and the error of low-expression genes can also easily become larger. It is also impossible to compare the difference between high-expression genes and low-expression genes; the ratios of 0.01/0.001 and 100/10 are the same while it is apparent that the later target gene may be more important. Therefore, it would be useful to provide researchers with a protocol for the relative quantification of genes, making the related analysis simpler and more accurate.

**Figure 1:**
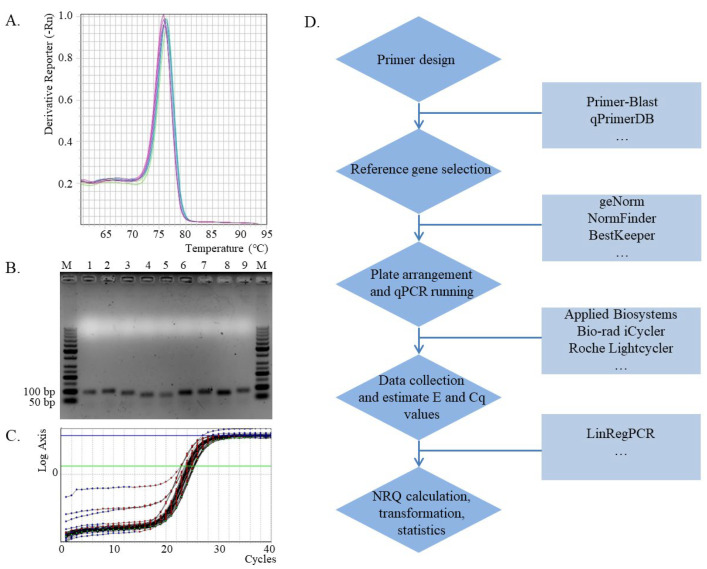
qPCR analysis and the workflow. (**A**). Melt curve for PCR products amplified from specific primers (*GAPDH2*) [6]. (**B**). Agarose gel electrophoresis detection of PCR products for the specificity of primers, M: DNA ladder; 1: *UBE2*; 2: *EF2*; 3: *β-TUB6*; 4: *snoR14*; 5: *snoR23*; 6: *ADP*; 7: *GAPDH1*; 8: *GAPDH2*; 9: *ACT* [6,10]. (**C**). Amplification efficiency and Cq calculation by LinRegPCR software (Rn data input for *GAPDH2*). (**D**). The workflow for qPCR analysis.

Previously, Rieu and Powers [4] provided a good reference in the calculation and statistics of qPCR analysis. Through years of work, the whole technique has also been refined, including primer design and reference gene selection [5,6,7,8,9]. Here, a workflow for qPCR analysis was proposed (Figure 1), and it will be beneficial to the study of gene expressions, especially for beginners. We further elaborate on the relative quantitative PCR in detail based on our recently published work (it is mainly about integrated analysis of transcriptome and metabolome under salt stress in rice) [9].

## 2. Primer Design and Validation

In terms of primer design, it is recommended to use the Primer-Blast software [11] available on the NCBI website, which can not only analyze the characteristics of the primers themselves, but also visually determine their potential binding sites and possible products in order to select specific primers. Of course, it is also possible to use primers provided directly in other researches, including systematic, professionally developed quantitative PCR primers such as those by Lu et al. [12] (https://biodb.swu.edu.cn/qprimerdb/, accessed on 1 July 2021). The primers of target genes were directly obtained from this web database for qPCR analysis in rice [9]. However, it is still advisable to conduct BLAST of the product sequences obtained from these primers in NCBI to determine their specificity.

For the specificity of these primers, a melt curve should first be conducted to determine whether a single peak indicating good specificity is obtained (Figure 1A). The PCR product should then be analyzed by electrophoresis with a 1.5% agarose gel, and if a single band is obtained, the specificity of the primers is confirmed (Figure 1B). If there is a high requirement for certainty, PCR products can also be sequenced to confirm primer specificity in further.

Other important requirements for primers are the Tm (melting temperature) value and amplicon size. The Tm value is best when it is close to 60 °C so that different primers can be used together, and it is often not necessary to set the extension temperature separately, but annealing and extension are completed in one step. The amplicon should preferably be between 75 and 150 bp, and appropriate lengthening is also possible, but not more than 250 bp.

## 3. Reference Gene Selection

It has been reported that the expression of some commonly used housekeeping genes may be altered by experimental treatments, so there may be risks in using commonly utilized housekeeping genes as reference genes [13]. Thus, it is better to use multiple reference genes for gene quantification. Recently, a lot of research work on reference gene selection has emerged, and many software packages have been developed, such as geNorm (v3.4) [14], NormFinder (v20) [15], BestKeeper (v1) [16]. Here, it is recommended to use the geNorm software because it can determine not only the stability of the reference genes, but also the least number of reference genes for quantitative analysis. Of course, it is also possible to directly obtain the corresponding best reference gene combination from other researchers’ work on reference gene selection. For salt treated rice seedling samples, two reference genes of *Os18S* and *Os25S* estimated by geNorm software were directly chosen from Jain et al. for normalization [17].

Considering that there is a lot of work on transcriptomic analyses, it is often necessary to use quantitative PCR to validate RNA-seq results, in which case the genes that have been shown to be unchanged in the transcriptome analysis can also be used as candidate reference genes [6].

## 4. Experimental Procedure

The total RNA of all samples is extracted and then the first-strand cDNA is synthesized, and the cDNA is usually diluted 10-fold for qPCR templates. The cDNA samples are also checked if there were DNA contaminations before qPCR amplification. The specific PCR primers, which anneal to sites flanking an intron within one gene, are designed. For rice experiments, primers of TTTCACTCTTGGTGTGAAGCAGAT and GACTTCCTTCACGATTTCATCGTAA for the eEF-1a gene were used [17]. The relative gene expression refers to the expression of the target gene relative to the reference genes, or the expression of the target gene is normalized by the reference genes. The PCR reactions should include all samples and non-template controls (NTC) for target genes and reference genes, respectively.

We can usually buy a kind of master mix containing SYBR Green dyes, and then take the appropriate mix and primers to configure a new PCR reaction mixture according to the manufacturer’s instructions, and then add the templates, while for NTC, only the addition of water is needed. Reaction conditions usually include an initial denaturation of 95 for 2 min, 40 cycles of denaturation at 95 °C for 15 s, annealing and extension at 60 °C for 1 min. If the annealing temperature is below 60 °C, it is necessary to separate the annealing and the extension temperature, as well as set the extension temperature to 72 °C. Fluorescence acquisition is performed in the extension step. Of course, there is a little bit of variation for the reaction condition, mainly due to the use of different types of instruments and different master mixes.

The above is mainly about the two-step qPCR method, and, of course, the one-step qPCR that directly uses RNAs as samples, which requires setting “No RT control” as the negative control. However, we do not recommend it here because RNA samples are very unstable and can also increase the complexity of the experimental operation.

## 5. Calculation

As the gene expression quantification, the NRQ (normalized relative quantity) can be calculated using this formula:

$$\mathrm{NRQ}=E_{Target\;gene}^{-\mathrm{Cq},\;Target\;gene}{/}^{n}\sqrt[{}]{E_{Reference\;gene\;1}^{-\mathrm{Cq},\;Reference\;gene\;1}\ldots E_{Reference\;gne\;n}^{-\mathrm{Cq},\;Reference\;gene\;n}}.$$

This calculation formula, which directly uses the actual value of PCR amplification efficiency (*E*) (*E* = 1 + e), does not require the *E* to be close to 2, resulting in a large increase in the available primers. We know that there are two common methods for estimation of PCR amplification efficiency, one through analysis of a dilution curve, the other by analysis of amplification curves of all reactions [18,19]. The *E* values can be calculated directly by the LinRegPCR software based on the Rn data of each reaction (or each well of the plate) from qPCR data without series of dilutions [18] (Figure 1C), and Cq (the quantification cycle) values can also be obtained at the same time. It can be determined that the *E* values of the same primer may vary greatly among different reactions. Theoretically, the same primer should have the same *E* value, so it is precise to use the mean of *E* values for all reactions while excluding abnormal *E* values and their corresponding Cq values [20].

In the rice salt tolerance experiment, the above formula can be further simplified to

$$\mathrm{NRQ}=E_{Target\;gene}^{-\mathrm{Cq},\;Target\;gene}/\sqrt[{}]{E_{Os18S}^{-\mathrm{Cq},\;Os18S}\cdot E_{Os25S}^{-\mathrm{Cq},\;Os25S}}.$$

The relative expressions of the target gene are presented by NRQ data, so the mean of NRQs (or $\bar{\mathrm{NRQ}}$) and corresponding standard errors (*Se*) (or $\bar{\mathrm{NRQ}}$ ± *Se*) are used to display the relative expressions of the target gene of each sample.

## 6. Statistics

For statistics, experimental design should be the starting point, such as the inclusion of at least three biological replicates for each sample and 2–3 technical replicates for each PCR reaction, ensuring that all biological replicates of all samples are on the same plate. When the experiment has a lot of treatments and one plate can no longer fit all the samples, multiple plates are needed, and each plate is best placed with one biological replicate of all samples; each plate can be regarded as a statistical block. Then, the NRQ data need to be transformed according to formula [Cq′ = log_2_(NRQ)] so that the Cq′ values and the Cq values are on the same scale [4]. The Cq′ value can be calculated for each biological replicate of each sample. Analysis of variance (ANOVA) can then be used to compare treatments using these Cq′ values of different treatments. The least significant difference (LSD) method can be used to compare differences at a particular level of significance (0.05 or 0.01 level), and this reduces to a *t* test if only two samples are compared.

## 7. Conclusions

In order to make qPCR analysis more scientific and accurate, this workflow of qPCR has been established (Figure 1D), which will provide benefits for the work in gene expression research. Prior to this workflow, total RNAs need to be extracted and then reverse-transcribed into cDNAs as templates for PCR reactions. Real-time qPCR analysis then needs to be completed according to this workflow, and changes in gene expression can be better understood. This workflow will show great advantages in transcriptome data validation. However, this method can only be used for relative gene expressions.

## Data Availability

Data is contained within the article.
